# Salad Vegetables as a Reservoir of Antimicrobial-Resistant *Enterococcus*: Exploring Diversity, Resistome, Virulence, and Plasmid Dynamics

**DOI:** 10.3390/foods14071150

**Published:** 2025-03-26

**Authors:** Ihab Habib, Mushtaq Khan, Glindya Bhagya Lakshmi, Mohamed-Yousif Ibrahim Mohamed, Akela Ghazawi, Rami H. Al-Rifai

**Affiliations:** 1 Veterinary Public Health Research Laboratory, Department of Veterinary Medicine, College of Agriculture and Veterinary Medicine, United Arab Emirates University, Al Ain P.O. Box 15551, United Arab Emirates; glindya_l@uaeu.ac.ae (G.B.L.); mohamed-yousif-i@uaeu.ac.ae (M.-Y.I.M.); 2 ASPIRE Research Institute for Food Security in the Drylands (ARIFSID), United Arab Emirates University, Al Ain P.O. Box 15551, United Arab Emirates; 3 Department of Medical Microbiology and Immunology, College of Medicine and Health Sciences, United Arab Emirates University, Al Ain P.O. Box 15551, United Arab Emirates; mushtaq.khan@uaeu.ac.ae (M.K.); akelag@uaeu.ac.ae (A.G.); 4 Zayed Center for Health Sciences, United Arab Emirates University, Al Ain P.O. Box 15551, United Arab Emirates; rrifai@uaeu.ac.ae; 5 Infectious Diseases Epidemiology Research Advancement Unit (IDERA), Institute of Public Health, College of Medicine and Health Sciences, United Arab Emirates University, Al Ain P.O. Box 15551, United Arab Emirates

**Keywords:** *Enterococci*, foodborne antimicrobial resistance, fresh produce, food safety, United Arab Emirates

## Abstract

This study investigates the occurrence, antimicrobial resistance (AMR) profiles, virulence factors, and plasmid composition of *Enterococcus* species isolated from salad ingredients in the United Arab Emirates (UAE). Four hundred salad vegetable items collected from local markets, over ten months through 2023, were screened, yielding an *Enterococcus* detection rate of 85.5% (342/400). *E. casseliflavus* was the most commonly identified species (50%), followed by *E. faecium* (20%) and *E. faecalis* (16%). Among 85 *Enterococcus* isolates tested for antimicrobial susceptibility, 55.3% displayed resistance to at least one agent, with 18.8% classified as multidrug-resistant (MDR). All isolates were not resistant to ampicillin, linezolid, teicoplanin, tigecycline, and high-level gentamicin. Intrinsic phenotypic resistance to vancomycin was found in *E. gallinarum* and *E. casseliflavus*, while low-level (<5%) ciprofloxacin and erythromycin resistance was sporadically detected in *E. faecium* and *E. faecalis*. Whole-genome sequencing (WGS) of 14 isolates (nine *E. faecium*, four *E. faecalis*, and one *E. casseliflavus*) unveiled a complex resistome. We report the first detection in salad vegetables of vancomycin resistance genes (*vanC*, *vanXY-C2*) in a vancomycin-susceptible *E. faecalis* isolate. Identifying *tetM*, *ermB*, and *optrA* genes in the studied isolates further underscored emerging resistance to tetracyclines, macrolides, and oxazolidinones. Concurrently, virulence gene analysis revealed 74 putative virulence factors, with *E. faecalis* harboring a higher diversity of biofilm-related and exoenzyme-encoding genes. One *E. faecalis* strain carried the cytolysin cluster (*cylI*, *cylS*, *cylM*), highlighting its pathogenic potential. Plasmid profiling identified 19 distinct plasmids, ranging from 3845 bp to 133,159 bp. Among the genome-sequenced isolates, mobilizable plasmids (47.3%) commonly carried AMR genes, especially *tet*(*L*) and *tet*(*M*), whereas conjugative plasmids (10.5%) did not harbor resistance determinants. These findings highlight that salad vegetables can still harbor and potentially transmit *Enterococcus* strains with clinically relevant resistance determinants and virulence traits. Enhancing foodborne AMR surveillance with WGS and targeted interventions is key to controlling its spread in the food.

## 1. Introduction

Salad vegetables are a vital component of a balanced meal and are highly valued for their numerous nutritional advantages. However, they are susceptible to microbial contamination from contact with soil, animal manure, contaminated irrigated water, and other environmental sources [1]. Thus, monitoring the occurrence and diversity of foodborne pathogens and hygienic bacterial indicator bacteria, including *Enterococcus* species, is critical to safeguarding consumers [2]. While *Enterococcus* species typically cause infections only under certain conditions, they have emerged as among the leading contributors to nosocomial infections in humans [2,3]. Among the various *Enterococcus* species identified in food and clinical-related samples, *Enterococcus faecalis* and *Enterococcus faecium* are the most prevalent [3]. The threat posed by species other than faecium and faecalis (e.g., *Enterococcus gallinarum* (particularly in immunocompromised individuals) and *Enterococcus casseliflavus* (linked to opportunistic infections, including bacteremia and urinary tract infections)) should not be overlooked, as these bacteria are also linked to human health [4,5].

*Enterococcus* species exhibit inherent resistance to a range of antibiotics, creating significant challenges for treatment on a global scale [3]. The degree of resistance varies by species, but they are inherently resistant to low-dose aminoglycosides, cephalosporins, clindamycin, carbapenems, polymyxins, and lincomycin [4,5]. Research investigating the resistance profiles of *Enterococcus* species has confirmed a global rise in multidrug-resistant strains, with a significant prevalence of resistance to vancomycin and tetracyclines [4]. It is worth mentioning that vancomycin-resistant *E. faecium* is included among the World Health Organization Bacterial Priority Pathogens List, 2024, particularly due to its ability to transmit resistance elements across the One Health spectrum [5]. A key factor in *Enterococcus* species adaptability is their highly flexible genome, enabling them to integrate foreign mobile genetic elements (e.g., plasmids) that harbor resistance determinants through horizontal gene transfer [6]. This highlights the need to monitor antimicrobial resistance in *Enterococcus* species, especially in the food chain, including salad vegetables typically consumed with minimal heat treatment or even raw, representing a potential threat of transmitting resistant bacteria to humans.

Understanding the genomic landscape of *Enterococcus* in raw vegetables is crucial for assessing the risks associated with consuming minimally processed foods and tracking the potential role of the global fresh produce trade in disseminating resistant bacteria [7,8]. Hence, this study investigates the diversity, antibiotic resistance profiles, putative virulence, and plasmid composition of *Enterococcus* species in everyday salad green items from the United Arab Emirates (UAE). Considering that a substantial amount of the UAE’s fresh produce and salad vegetables are sourced from imports, this research carries significant implications beyond the local context. By employing whole-genome sequencing (WGS) and bioinformatic mining of genomic data, this research hopes to generate a comprehensive picture of the resistome, virulome, and plasmid architecture of *Enterococcus* strains in fresh produce. The novelty of this work lies in providing the first comprehensive genomic characterization of *Enterococcus* species isolated from salad vegetables consumed raw or minimally processed in the UAE context. The insights gained from this study can strengthen food safety policies, enhance surveillance frameworks, and inform global efforts to curb the propagation of antimicrobial resistance across the food supply.

## 2. Materials and Methods

### 2.1. Sources and Sample Selection

A structured sampling approach was implemented by purchasing fresh salad vegetables from retail outlets. The sample size was determined using a presumed detection rate of 50%, a 95% certainty level, and a 5% allowable error [9]. Four hundred samples were gathered and analyzed year-round throughout 2023, excluding July and August due to the summer break. The sampling covered major communal markets (n = 6) and major supermarkets (n = 46) in Dubai, Abu Dhabi, and Al-Ain cities, ensuring a representative selection of locally produced (72.5% of the samples) and imported (27.5% of the samples) items. To maintain sample integrity, only vegetables free from visible dirt and spoilage were selected. Samples were stored refrigerated at 4 °C until testing commenced, wrapped in sterile material, and transported for laboratory analysis on the same collection day.

### 2.2. Enterococcus Isolation and Characterization

In the course of microbiological testing, 10 g of each sample was blended with 90 mL of Buffered Peptone Water (Oxoid, Basingstoke, UK) for two minutes (BagMixer^®^ 400 P, Interscience, Saint Nom la Brétèche, France). This initial mixture was labeled 10^−1^, and a 10^−2^ dilution was subsequently prepared using 0.1% peptone water. From both dilutions (10^−1^ and 10^−2^), 100 µL was plated onto Slanetz–Bartley agar (Oxoid, UK) and incubated for 36–48 h at 41.5 °C [10]. Following incubation, as many as five colonies per plate that showed typical *Enterococcus* characteristics were chosen for species confirmation using the Autobio ms1000 MALDI-TOF system (Autobio Diagnostics, Zhengzhou, China). According to the manufacturer’s protocols, identification scores falling above 9.000 were regarded as reliable for species-level classification.

### 2.3. Evaluation of Phenotypic AMR

Eighty-five strains representing four *Enterococcus* species were assessed for their antimicrobial susceptibility profiles using the Vitek-2 automated platform (bioMérieux, Craponne, France). The AST-P592 panel (bioMérieux) was used to test resistance against ten antibiotics: ampicillin, erythromycin, ciprofloxacin, high-level gentamicin, high-level streptomycin, tigecycline, teicoplanin, tetracycline, vancomycin, and linezolid [10]. Classification of isolates as susceptible, intermediate, or resistant followed the manufacturer’s built-in classification, utilizing CLSI standards [11]. Isolates showing resistance to greater than one agent in ≥3 antimicrobial groups were identified as multidrug-resistant (MDR) [12].

### 2.4. Whole-Genome Sequencing

WGS was conducted on nine *E. faecium*, four *E. faecalis*, and one *E. casseliflavus*, chosen for their notable phenotypic resistance characteristics. The library preparation and sequencing were executed through the service provider Novogene (Cambridge, UK) using the HiSeq platform (Illumina, San Diego, CA, USA). Subsequent analyses were performed through the Solu platform (Solu Healthcare Inc., Helsinki, Finland), enabling batch processing for species determination, multilocus sequence typing (MLST), resistance genes and mutations, and putative virulence determinants [13]. Gene identification employed a ≥95% sequence identity threshold and a minimum 60% sequence coverage cutoff [14]. The raw read data have been deposited in the National Library of Medicine (NCBI) under Bio-Project: PRJNA1231383.

### 2.5. Plasmid Mobility Evaluation

Plasmid transfer capabilities were investigated using MOB-suite (v3.1.0), which analyzes assembled contigs to detect key plasmid-related features such as relaxase and replicase genes, as well as repetitive regions [15]. The identified plasmid sequences are then matched against a reference database of mobility clusters (MOB-clusters) by calculating the minimal Mash distance, allowing putative plasmids to be grouped into specific MOB-clusters. These plasmids are subsequently labeled as “Conjugative”, “Mobilizable”, or “Non-mobilizable” based on their relaxase gene profiles [16]. The relaxase genes recognize and cleave the origin of transfer (oriT), thereby facilitating horizontal gene transfer and, consequently, the spread of genes conferring resistance and virulence attributes among bacteria [15,16]. After running the MOB-suite on all assemblies, the resulting data were consolidated and examined using the Solu cloud platform, which provides real-time genomic pathogen monitoring [13,15].

## 3. Results

### 3.1. Diversity of Enterococcus Species

**Figure 1:**
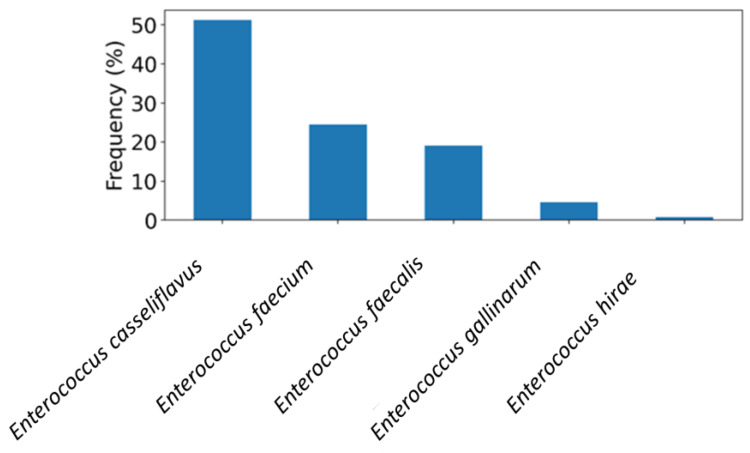
The frequency distribution of *Entercoccus* species recovered from salad vegetable samples (n = 400) in the UAE.

Four hundred salad vegetable samples obtained from retail markets in the UAE were tested to assess the occurrence of *Enterococcus* species. The rate of detection was 85.5% (342/400), with *E. casseliflavus* emerging as the most abundant species (50%, n = 171) (Figure 1). *E. faecium* and *E. faecalis* were identified in 20% and 16% of the *Enterococcus*-positive samples, respectively (Figure 1). Additional species were *E. gallinarum* and *E. hirae*, detected at lower frequencies as illustrated in Figure 1.

### 3.2. Antimicrobial Susceptibility Profiles Across Enterococcus Species

**Figure 2:**
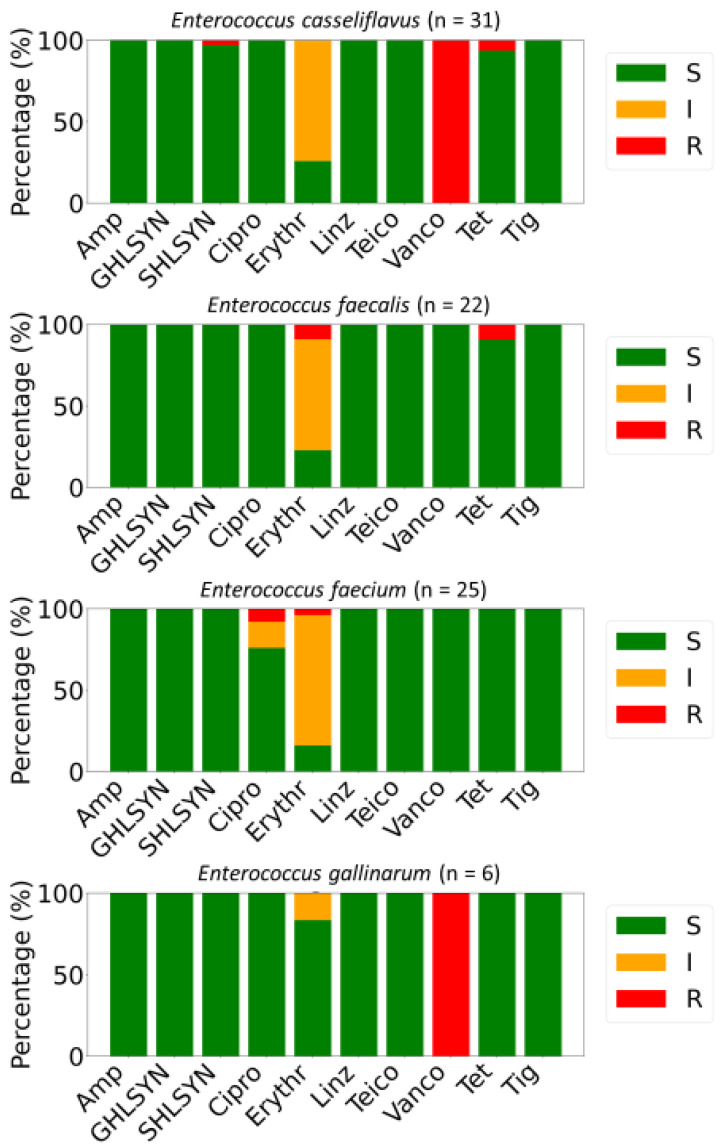
Distributions of antimicrobially resistant (R, red), susceptible (S, green), and intermediate (I, orange) isolates among *Enterococcus* spp. isolated from salad greens in the UAE. The panel of antimicrobials included ciprofloxacin (Cipro), tetracycline (Tet), ampicillin (Amp), erythromycin (Erythr), high-level gentamicin (GHLSYN), streptomycin (SHLSYN), vancomycin (Vanco), tigecycline (Tig), teicoplanin (Teico), and linezolid (Linz).

Antimicrobial susceptibility testing of 85 *Enterococcus* isolates demonstrated variability in resistance patterns among species. Non-susceptibility to a minimum of one antibiotic was observed in 55.3% (n = 47) of the isolates, while 18.8% (n = 16) were classified as MDR (Figure 2). All isolates exhibited full susceptibility to ampicillin, high-level gentamicin (GHLSYN), linezolid (Linz), teicoplanin (Teico), and tigecycline (Tig). Intrinsic resistance toward vancomycin was detected in all *E. gallinarum* and *E. casseliflavus* isolates. High-level streptomycin (SHLSYN) resistance was identified in 1.18% of strains, exclusively among *E. casseliflavus*, while 98.82% remained susceptible. Resistance to ciprofloxacin was revealed in 2.35% of the characterized *E. faecium* strains, with 4.71% displaying intermediate resistance. Additionally, 3.53% of strains were resistant to erythromycin, whereas a substantial proportion (69.41%) exhibited intermediate susceptibility to this antibiotic (Figure 2).

### 3.3. Whole-Genome Analysis: Resistome Composition

**Figure 3:**
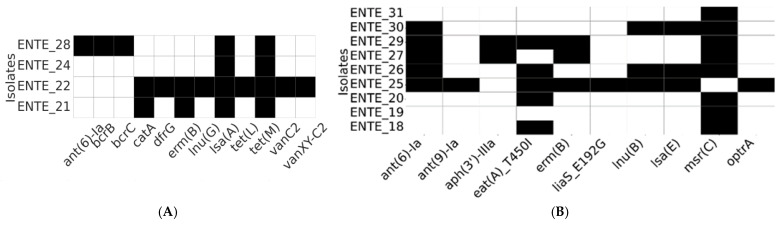
Resistome profiles of the whole-genome-sequenced *E. faecalis* (**A**) and *E. faecium* (**B**). The heatmap depicts the presence (black) or absence (white) of antimicrobial resistance determinants.

WGS was performed on nine *E. faecium* and four *E. faecalis* isolates, selected based on their phenotypic resistance profiles. The resistome analysis identified diverse antimicrobial resistance genes (Figure 3). We also sequenced one *E. casseliflavus*, where the only AMR genes found in it were those associated with inherent vancomycin resistance (*vanC*, *vanR-C*, *vanS-C*, *vanTc*, and *vanXY-C*), and all were predicted to be chromosomal.

Vancomycin resistance genes (*vanC*, *vanXY-C2*; chromosome located) were detected in a vancomycin-susceptible *E. faecalis* strain (ENTE-22) recovered from a romaine lettuce sample imported from Jordan. Similarly, the *optrA* gene (predicted to be chromosome-located), associated with oxazolidinone resistance, was detected in a linezolid-susceptible *E. faecium* isolate originating from a UAE-grown romaine lettuce sample (Figure 3).

Among the identified resistance determinants, the tetracycline resistance gene (*tetM*) was exclusively present in all *E. faecalis* isolates. Additionally, *lsa*(*A*), a gene responsible for resistance to lincosamides and streptogramins, was found in every *E. faecalis* isolate. The *msr*(*C*) gene, which mediates macrolide resistance, was widely distributed except for a single *E. faecium* isolate. Genes conferring aminoglycoside resistance (*ant*(*6*)-*Ia*) and macrolide resistance (*ermB*) were shared among both *E. faecalis* and *E. faecium* (Figure 3).

### 3.4. Whole-Genome Analysis: Virulome Composition

**Figure 4:**
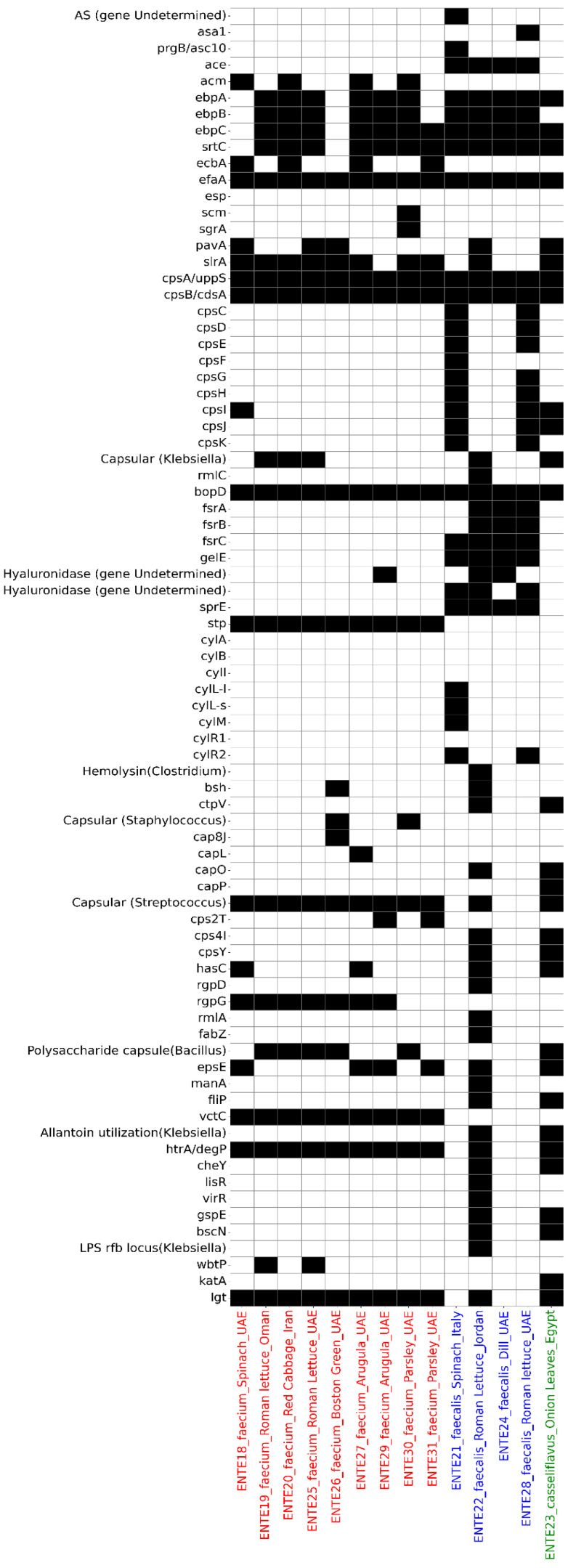
The putative virulome profile of the whole-genome-sequenced *E. faecium* (labeled red on the *x*-axis), *E. faecalis* (labeled blue on the *x*-axis), and *E. casseliflavus* (labeled green on the *x*-axis). The heatmap depicts the presence (black) or absence (white) of antimicrobial resistance determinants.

Virulence gene analysis identified 74 putative virulence genes across the 14 sequenced isolates, with the sum of putative virulence genes per isolate ranging from 17 (ENTE-18, *E. faecium*) to 44 (ENTE-22, *E. faecalis*) (Figure 4). The sequenced *E. casseliflavus* also harbored 34 putative virulence genes (Figure 4).

The most frequently detected virulence-associated genes were those encoding adherence factors, with *E. faecalis* carrying the *ace* gene in 100% of isolates. The *efaA* gene was universally present across all 14 isolates. Genes associated with biofilm formation, such as those from the *fsr* operon, were exclusively found in *E. faecalis* isolates, while they were absent in *E. faecium* and *E. casseliflavus* (Figure 4).

Several exoenzyme genes were detected, including gelatinase (*gelE*), presented in all *E. faecalis* isolates, and hyaluronidase, identified in three isolates (two *E. faecalis* and one *E. faecium*). Toxin-associated genes varied between isolates. The *stp* gene, encoding serine/threonine phosphatase, was present in all *E. faecium* isolates, while *sprE*, encoding a metalloprotease enzyme, was identified in all *E. faecalis* isolates. The *cyl* cluster of genes (*cylI*, *cylS*, *cylM*), responsible for cytolysin production, was evident in only one *E. faecalis* isolate, originating from spinach imported from Italy (Figure 4).

The *htrA*/*degP* gene, which plays a critical role in bacterial stress response and survival, was found in all isolates except three *E. faecalis* isolates. Genes encoding the putative type-II secretion system (*gspE*) and type-III secretion system (*bscN*) were also identified in one *E. faecalis* and one *E. casseliflavus* isolate. These isolates also carried *filP*, a gene associated with bacterial invasion and cytoskeletal-like protein synthesis (Figure 4).

### 3.5. Plasmid Architecture of Enterococcus Isolates

Using MOB-suite bioinformatics tools, plasmid profiling identified 19 distinct plasmids among the sequenced *Enterococcus* isolates. Plasmid sizes ranged from 3845 bp to 133,159 bp, with the number of plasmids varying from one to four per isolate (Table 1).

*E. faecium* isolates exhibited a greater plasmid diversity than *E. faecalis*, with replicon typing revealing predominant clusters such as rep_cluster_185, rep_cluster_893, and rep_cluster_1018 (Table 1). Mobility classification categorized plasmids into mobilizable (47.3%), non-mobilizable (42.2%), and conjugative (10.5%) groups. The results in Table 1 indicate that mobilizable plasmids frequently harbored ARGs, particularly *tet*(*L*) and (*M*), which confer resistance to tetracycline. In contrast, conjugative plasmids, predicted in two isolates, did not carry any antimicrobial resistance genes (Table 1).

## 4. Discussion

In recent years, enterococci have continued to emerge as opportunistic pathogens and a significant cause of nosocomial infections due to their efficient host adaptation and ability to acquire virulence and antibiotic-resistance genes [2,17]. The high prevalence of *Enterococcus* spp. in salad vegetables observed in this study underscores fresh produce’s potential role in transmitting antimicrobial-resistant bacteria to consumers. The detection rate of 85.5% revealed in this study aligns with previous reports from Oman [18], Korea [19], and Poland [20] that have documented the widespread occurrence of *Enterococcus* species in fresh produce. Researchers studying plant-derived foods suggest that these products create an environment where enterococci are highly prevalent, mainly due to contact with soil and fertilizers [19]. The predominance of *E. casseliflavus* over clinically significant species such as *E. faecalis* and *E. faecium* warrants further investigation, as this species, though typically considered of lower pathogenic potential, has been associated with intrinsic resistance to vancomycin [21].

The antimicrobial susceptibility profiles for isolates in this study revealed variable resistance among *Enterococcus* species, with intrinsic vancomycin resistance detected widely in *E. casseliflavus* and *E. gallinarum* isolates. This finding was consistent with reports from Oman [18], Poland [20], and Tunisia [22], where these species have well-known chromosomally encoded intrinsic *vanC*-mediated resistance [23]. Our study reports the first detection in *E. faecalis* from salad vegetables (Roman lettuce, imported from Jordan) of vancomycin resistance genes (*vanC*, *vanXY-C2*) in a vancomycin-susceptible isolate. Even if resistance is not expressed in this isolate, our finding highlights that *E. faecalis* can acquire resistance genes, potentially from co-inhabiting species within the same food/agriculture ecosystem, that carry them intrinsically (e.g., from the widespread *E. casseliflavus* in fresh produce) [24,25]. This emphasizes the need for ongoing surveillance of this species. Similarly, identifying *optrA* in linezolid-susceptible *E. faecalis* isolates suggests the potential for silent reservoirs of resistance, where genes remain functionally inactive or require specific environmental triggers for expression [10]. Given the clinical significance of vancomycin and linezolid in treating enterococcal infections, surveillance for such latent resistance elements is imperative to mitigate future resistance emergence.

The absence of resistance to high-level gentamicin, linezolid, ampicillin, and tigecycline is encouraging, indicating that these antibiotics remain well preserved through effective stewardship and responsible agricultural practices [26]. However, the presence of MDR strains in 18.8% of isolates is a concern, as it reflects the potential for fresh salad greens to serve as reservoirs for antibiotic-resistant bacteria. Similar resistance patterns have been noted in studies in Oman and South Korea [18,19], highlighting this issue’s global nature. The detection of ciprofloxacin resistance (2.35%) among *E. faecium* isolates is particularly noteworthy, given the critical role of fluoroquinolones in treating enterococcal infections. In the USA, ciprofloxacin resistance was detected in 5% of *E. faecalis* and 28% of *E. faecium* isolates from fresh produce [27]. Notably, these rates of resistance are higher compared to our present study in the UAE, highlighting potential regional differences in resistance patterns to clinically relevant antimicrobials and the influence of local agricultural and environmental factors.

For the first time in the UAE and the Middle East, this study conducted comprehensive whole-genome bioinformatics analyses to examine antimicrobial resistance, plasmid content, and putative virulence genes in *Enterococcus* spp. from plant-derived foods. Except for one isolate, all *E. faecium* isolates studied have shown the presence of the chromosomal *msrC* gene, contributing to the intrinsic resistance to macrolide–streptogramin B [28]. Also, in *E. faecium*, the gene *aac*(*6*′)-*Ie-aph*(*2*″)-*Ia*, hypothesized to be associated with gentamicin resistance (HLGR), was widely presented; however, this was not translated to phenotypic resistance in any of the isolates. Previous research noted that some *E. faecium* strains with this gene do not exhibit HLGR, due to insertions like *IS1216V*, which disrupt the gene’s function and lead to a loss of the HLGR phenotype [29]. On the other hand, the *tetM* gene was found in all *E. faecalis* isolates characterized in this study and was associated with phenotypical resistance levels. Also, based on MOB-suite analysis [15], we pointed out that such a gene was linked in several isolates to mobile genetic elements (e.g., plasmids), a feature that increases the risk of *tetM* transfer to other bacterial species [30].

Plasmid-mediated AMR plays a crucial role in the persistence and dissemination of resistance genes in foodborne *Enterococcus* spp. [30]. This study identified a diverse array of plasmid replicons among isolates, highlighting the capacity of plant-derived isolates to maintain horizontal gene transfer, a critical mechanism in disseminating AMR determinants. The greater plasmid diversity observed in *E. faecium* isolates compared to *E. faecalis* is likely attributable to differences in their genomic plasticity and ecological adaptability. *E. faecium* is widely recognized for its highly flexible genome, enhancing its ability to acquire and maintain diverse mobile genetic elements, including plasmids. This adaptability may reflect *E. faecium*’s broader environmental niches, stronger selective pressure in various habitats, and higher frequency of horizontal gene transfer events. In contrast, *E. faecalis* generally exhibits a relatively more stable genome, reducing its capacity for extensive plasmid acquisition and diversity [27,30].

This study also revealed a widespread distribution of putative virulence-associated genes across *Enterococcus* isolates, including those linked to biofilm formation. These virulence factors enhance the ability of enterococcal isolates to acquire adaptive elements, providing evolutionary advantages that improve their survival and persistence in various environments [30]. While some virulence determinants, such as *gelE* (gelatinase) and *hyl* (hyaluronidase), have established roles in pathogenicity, many putative virulence genes remain poorly understood [31]. It should be noted that the detection of multiple virulence genes in non-clinical isolates suggests that their presence alone may not indicate pathogenic potential, reinforcing the need for functional validation studies [32]. Future work should investigate the expression and regulation of these genes in foodborne *Enterococcus* to better understand their role in bacterial fitness, persistence, and potential virulence within the food supply chain.

This study generates novel insights into the prevalence, antimicrobial resistance, and virulence profiles of *Enterococcus* spp. in fresh produce, offering the first comprehensive whole-genome bioinformatics analysis of these bacteria in plant-derived foods in the UAE and the Middle East. However, some limitations should be acknowledged. This study was conducted on a specific set of fresh produce samples, which may not fully capture the broader diversity of *Enterococcus* contamination across different food types and seasons. Additionally, while genetic analyses revealed key antimicrobial resistance and virulence determinants, functional validation of these genes was beyond the scope of this study. Despite these constraints, the findings lay a strong foundation for future research. Future investigations should also explore the impact of agricultural practices and environment on the dissemination of *Enterococcus* in fresh produce, strengthening food safety strategies from a One Health perspective.

## 5. Conclusions

To our knowledge, this study presents the first reported *Enterococcus* spp. genome sequences isolated from fresh salad greens in the UAE and Middle East. This work highlights the need for enhanced surveillance of *Enterococcus* spp. in fresh produce, particularly in settings dependent on imported food, such as in the UAE. The detection of MDR strains, including those with intrinsic vancomycin resistance, underscores the importance of stricter regulations on antimicrobial use in agriculture. A multidisciplinary approach integrating microbiological surveillance, genomic epidemiology, and One Health policies is crucial to mitigating AMR risks. WGS should be utilized to assess the interplay between resistance and virulence, informing food safety policies and risk assessments. Stricter monitoring is essential to prevent foodborne *Enterococcus* from becoming reservoirs of clinically relevant resistance genes. To effectively address these findings, regulatory and monitoring agencies should implement targeted screening protocols for *Enterococcus* spp. in imported produce, integrating WGS into routine surveillance programs to rapidly identify and trace AMR strains. Enhancing international collaboration and data-sharing frameworks can further improve tracking and managing risks associated with antimicrobial-resistant pathogens in global food trade. Additionally, educational initiatives and training for agricultural producers regarding antimicrobial stewardship and hygiene practices can significantly contribute to reducing contamination risks at the source.

## Figures and Tables

**Table 1:** Plasmid diversity, mobility, and replicon type analysis of 19 predicted plasmids from the whole-genome analysis of *Enterococcus* isolated from salad vegetable samples in the UAE.

Species	Isolates	Source	No. of Plasmids	Size (bp)	MOB Cluster	Resistance Genes Harbored on Plasmid	Replicon Types	Mobility
*Enterococcus faecium*	ENT-31	Parsley UAE	1	26,145	AB756	*tet*(*L*), *tet*(*M*)	rep_cluster_1018, rep_cluster_185	mobilizable
	ENT-30	Parsley UAE	3	52,765	AC731	None	rep_cluster_893	non-mobilizable
				27,735	AB756	*ant*(*6*)-*Ia*, *lnu*(*B*), *lsa*(*E*), *spw*, *tet*(*L*), *tet*(*M*)	rep_cluster_185	mobilizable
				5972	AA894	None	rep_cluster_1742	mobilizable
	ENT-29	Arugula, UAE	2	133,159	AD908	None	rep_cluster_893	conjugative
				44,140	AB756	*ant*(*6*)-*Ia*, *aph*(*3*′)-*IIIa*, *erm*(*B*), *sat4*, *tet*(*L*), *tet*(*M*)	rep_cluster_1018, rep_cluster_185	mobilizable
	ENT-27	Arugula, UAE	2	116,750	AC727	None	Inc18, rep_cluster_893	non-mobilizable
				19,166	AB756	*tet*(*L*), *tet*(*M*)	rep_cluster_1018, rep_cluster_185	mobilizable
	ENT-26	Boston Green Lettuce, UAE	3	81,412	AC727	None	rep_cluster_893	non-mobilizable
				30,390	AD582	*tet*(*L*), *tet*(*M*)		mobilizable
				18,377	AB918	*ant*(*6*)-*Ia*, *lnu*(*B*), *lsa*(*E*), *spw*		non-mobilizable
	ENT-25	Roman Lettuce, UAE	4	48,220	AD582	*tet*(*L*), *tet*(*M*)		mobilizable
				40,528	AD907	None	rep_cluster_893	conjugative
				10,144	AB915	*erm*(*B*)		non-mobilizable
				51,220	AC728	None		mobilizable
*Enterococcus faecalis*	ENT-28	Roman Lettuce, UAE	1	3845	AD569	None	rep_cluster_1197	non-mobilizable
	ENT-22	Roman Lettuce, Jordan	2	65,530	AD582	*catA*, *dfrG*, *tet*(*L*), *tet*(*M*)	rep_cluster_1118, rep_cluster_992	mobilizable
				6362	AD265	None		non-mobilizable
	ENT-21	Spinach, Italy	1	5982	AD058	*catA*, *erm*(*B*)	rep_cluster_1118	non-mobilizable

## Data Availability

The original contributions presented in this study are included in the article. Further inquiries can be directed to the corresponding author. The raw read data have been deposited in the National Library of Medicine (NCBI) under Bio-Project: PRJNA1231383.
